# Characterizing ceftriaxone tolerance in *Neisseria gonorrhoeae* across *in vitro* and *in vivo* models

**DOI:** 10.1128/msystems.01298-25

**Published:** 2026-01-08

**Authors:** Izumo Kanesaka, Anurag Kumar Bari, Saïd Abdellati, Thibaut Vanbaelen, Irith De Baetselier, Tessa de Block, Reinout Naesens, Basil Britto Xavier, John Rossen, Chris Kenyon, Sheeba Santhini Manoharan-Basil

**Affiliations:** 1 Department of Clinical Sciences, Institute of Tropical Medicine Antwerp 37463 , Antwerp, Belgium; 2 Department of Infection Control and Prevention, Faculty of Nursing, Toho University 13103, Tokyo, Japan; 3 Department of Medical Microbiology and Infection Prevention, Antimicrobial Resistance, Genomics and Epidemiology (AGE) Research Group, University Medical Center Groningen, University of Groningen 3647, Groningen, the Netherlands; 4 Department of Clinical Biology, ZAS, Antwerp, Belgium; 5 Laboratory of Medical Microbiology and Infectious Diseases and Isala Academy, Isala Hospital, Zwolle, the Netherlands; 6 Department of Pathology, University of Utah School of Medicine 12348 , Salt Lake City, Utah, USA; 7 University of Cape Town 37716, Cape Town, South Africa; University of California, San Francisco, San Francisco, California, USA

**Keywords:** tolerance, *Neisseria gonorrhoeae*, ceftriaxone, MDK99, treatment failure, *Galleria mellonella*

## Abstract

This study aims to characterize the phenotypic behavior and *in vivo* persistence of a ceftriaxone-tolerant *Neisseria gonorrhoeae* clinical isolate from a single patient and evaluate the potential role of tolerance in treatment failure. A previously identified ceftriaxone-tolerant vaginal isolate was compared with isogenic and clinical non-tolerant strains. Bacterial growth was assessed *in vitro*, and tolerance was quantified using the minimum duration required to kill 99% of the population (MDK99), and persistence was evaluated in an *in vivo Galleria mellonella* infection model. Whole-genome sequencing (WGS) and transcriptomic (RNA-sequencing [RNA-seq]) profiling were performed to identify tolerance-associated genetic and transcriptional signatures. The tolerant strain exhibited prolonged MDK99 values across ceftriaxone concentrations, persisting for up to 24 hours under drug exposure. It also showed delayed early-phase growth, suggesting a fitness cost. *In vivo*, the tolerant strain remained viable up to 8 hours after treatment, whereas non-tolerant strains were cleared. WGS revealed identical gene content across all isolates, but non-synonymous mutations in pilE_3, a type IV pilin gene, were exclusively present in tolerant strains. RNA-seq analysis showed upregulation of pilin-associated genes and downregulation of zinc-independent ribosomal paralogs (*rpmE2* and *ykgO*), suggesting a combined mechanism of surface remodeling and translational suppression associated with the tolerant phenotype. Ceftriaxone tolerance enables prolonged survival of *N. gonorrhoeae* despite apparent susceptibility by standard MIC-based testing. This phenotype may contribute to treatment failure, recurrent infection, and ongoing transmission, indicating the need for revised diagnostic and therapeutic strategies.

**IMPORTANCE:**

Ceftriaxone remains the last reliable option for gonorrhea therapy, yet recurrent infections can occur despite isolates being classified as susceptible by MIC testing. One possible explanation is antibiotic tolerance, a phenotype that allows survival during drug exposure without changes in MIC. Although tolerance has been described in other pathogens, its role in gonococcal infection has remained poorly defined. In this study, we provide the first detailed characterization of a ceftriaxone-tolerant *Neisseria gonorrhoeae* clinical isolate associated with repeated treatment failure. By combining *in vitro* killing assays, an *in vivo Galleria mellonella* infection model, whole-genome sequencing, and transcriptomic profiling, we demonstrate that tolerance enables prolonged survival under ceftriaxone and is linked to pilin gene variation and ribosomal remodeling. These findings illustrate how a clinically observed phenomenon can be mechanistically dissected and emphasize tolerance as a hidden factor contributing to gonococcal persistence and potential treatment failure.

## INTRODUCTION

The increasing prevalence of antimicrobial-resistant *Neisseria gonorrhoeae* represents a serious global health challenge. While the mechanisms of acquired resistance, including β-lactamase production, penicillin-binding protein alterations, and efflux pump activation, have been extensively characterized, bacterial tolerance remains a less explored yet clinically significant phenomenon. Tolerance refers to a heritable or non-heritable phenotype wherein a bacterial subpopulation survives antibiotic exposure without an elevated minimum inhibitory concentration (MIC) (1). Unlike resistance, tolerance is not detected by standard antimicrobial susceptibility testing (AST) but may nonetheless contribute to unexplained treatment failures (2, 3). Growing evidence from other bacterial pathogens, such as *Staphylococcus aureus*, *Escherichia coli*, and *Mycobacterium tuberculosis*, has shown that tolerance facilitates bacterial persistence under antibiotic pressure, prolongs infection duration, and increases the risk of treatment failure and relapse (2, 3). Furthermore, tolerance has been implicated in driving the evolution of resistance, as tolerant subpopulations can survive antibiotic treatment, providing a reservoir of cells where resistance mutations may emerge and become selected (3–5). Despite its recognized role in other pathogens, the contribution of tolerance to persistent infections and resistance development in sexually transmitted infections (STIs), including gonorrhea, remains largely unexplored. This knowledge gap is concerning given the global burden of gonorrhea and the increasing threat of multidrug-resistant strains. The World Health Organization has classified *N. gonorrhoeae* as a high-priority pathogen for research and development of new treatment strategies (6). However, the potential role of tolerance in treatment failure, ongoing transmission, and accelerating resistance development has not been systematically investigated in this pathogen. Understanding tolerance in *N. gonorrhoeae* is therefore essential not only to address a major scientific knowledge gap but also to inform diagnostic, therapeutic, and surveillance strategies. Developing and applying robust methods to detect and characterize tolerance will be crucial for assessing its clinical and public health significance.

Previously, we have shown that tolerance can be induced in *N. gonorrhoeae* through repeated exposure to ceftriaxone, and transcriptomic analysis has revealed that ceftriaxone tolerance is associated with the downregulation of various ribosomal genes, suggesting a mechanism for bacterial survival under antibiotic stress (7). Recent studies have demonstrated the presence of ceftriaxone-tolerant *N. gonorrhoeae* isolates, particularly from urethral and rectal infections (7, 8). These findings suggest that tolerance may enable gonococci to persist under therapeutic antibiotic concentrations, leading to recurrent infections. Our previous case report was the first to document ceftriaxone tolerance in a vaginal *N. gonorrhoeae* isolate from a patient with repeated treatment failures despite confirmed susceptibility (9). This observation highlighted tolerance as a potential but underrecognized factor in gonococcal treatment failure in this and other cases (10, 11). However, several critical questions remain unanswered. While detection methods such as the tolerance detection (TD) test can identify tolerant phenotypes, the biological and clinical implications of tolerance in *N. gonorrhoeae* are poorly understood. The TD test is a modification of the standard disk-diffusion assay that enables the semiquantitative evaluation of antibiotic tolerance by promoting the growth of surviving bacteria within the inhibition zone after antibiotic diffusion (12). It is unknown how tolerance affects bacterial growth kinetics, fitness costs, and persistence under antimicrobial pressure. Without addressing these gaps, the role of tolerance in treatment failure remains speculative.

The aim of this study is to characterize the phenotypic behavior of a ceftriaxone-tolerant *N. gonorrhoeae* strain identified in our previous case report (9). By comparing this strain to an isogenic non-tolerant clone, we seek to elucidate how tolerance influences bacterial growth and survival under antibiotic exposure. These insights are essential for recognizing tolerance as a distinct factor in the management of gonococcal infections and for advancing the understanding of tolerance as a broader microbial survival strategy.

## MATERIALS AND METHODS

### Strains used in this study and TD

Four *N. gonorrhoeae* clinical isolates from a transgender man with recurrent gonococcal cervicitis were analyzed in this study (Table 1) (9). (i) Isolate 24903 was collected on 20 December 2024 from a vaginal swab during his fifth episode of cervicitis since August 2024. A ceftriaxone TD test revealed no evidence of tolerance. Each of his prior episodes of gonorrhea had been treated at various primary health care facilities with ceftriaxone 1 g intramuscular injection (IMI) with symptomatic improvement but then recurrence. His partner was also treated simultaneously. This isolate was obtained during his first visit to our clinic at the Institute of Tropical Medicine, Antwerp. (ii) His symptoms regressed after receipt of ceftriaxone on 20 December but had returned by 29 December 2024 when isolate No. 24929 was obtained from a cervical swab. This isolate had a slightly higher ceftriaxone MIC of 0.008 µg/mL compared to 24903 (0.002 µg/mL) and exhibited tolerance on the TD test (13). Both the tolerant and non-tolerant subpopulations of isolate 24929 showed the same MIC for ceftriaxone (0.008 µg/mL). The TD test was performed to identify ceftriaxone-tolerant subpopulations capable of surviving transient antibiotic exposure. In this assay, bacterial cultures were exposed to ceftriaxone under controlled conditions, washed to remove residual antibiotic, and reinoculated into antibiotic-free gonococcal (GC) broth supplemented with IsoVitaleX. Regrowth after overnight incubation indicated a tolerant phenotype (TD-positive), whereas its absence indicated TD-negative. This simple regrowth-based assay allows the detection of tolerance phenotypes that are not captured by standard MIC-based susceptibility testing and provides a practical screening approach for recurrent or treatment-failure cases. The procedure was based on the method described by Balduck et al. (8) with minor modifications to the incubation time and washing steps. He was once again treated with 1 g of ceftriaxone IMI and had initial improvement of symptoms but returned with symptomatic cervicitis and pelvic inflammatory disease (PID) on 19 January 2025. (iii) A cervical swab from this swab provided the third isolate, (25061; CRO- MIC 0.008 µg/mL). He was treated with ceftriaxone 1 g IMI, doxycycline PO 100 mg BID × 14 days and metronidazole PO 500 mg BID × 14 days. (iv) His symptoms resolved, but they returned in early February, and he represented at our clinic on 5 February 2025 with symptomatic cervicitis when the fourth isolate was obtained (25109; CRO- MIC 0.016 µg/mL). Both isolates 25061 and 25109 exhibited tolerance on the TD test. He was treated with a prolonged course of ceftriaxone (four doses of 1 g IMI over 7 days) and has not had recurrence of his cervicitis since this. A *N. gonorrhoeae* PCR (Roche Cobas 5800 [Roche Diagnostics, Branchburg, NJ, USA]) of the cervix, rectal, and pharyngeal swabs was also negative one month post-treatment.

**TABLE 1:** Characteristics of clinical *N. gonorrhoeae* isolates showing MIC tolerance phenotype and genotyping results

Strain ID	Isolation date (day/mo/yr)	Origin	MIC (µg/mL)			Treatment	TD test	MDK99	RNA-seq	WGS (DNA-seq)	Sequence type
			Ceftriaxone	Ciprofloxacin	Azithromycin						
24903	20/12/2024	Vaginal	0.002	0.006	1.5	CRO 1 g IMI	Negative	Yes	No	No	NA^*a*^
24929	29/12/2024	Vaginal	0.008	2	1	CRO 1 g IMI	Positive	Yes	Tolerant: yes	Tolerant: yes	ST7822
								Yes	Non-tolerant: yes	Non-tolerant: yes	ST7822
25061	19/01/2025	Vaginal	0.008	2	1.5	CRO 1 g IMI and 14 days of doxycycline and metronidazole	Positive	No	No	Yes	ST7822
25109	2/05/2025	Vaginal	0.016	>32	1.5	4 doses of CRO 1 g IMI over 7 days	Positive	No	No	Yes	ST7822

^*a*^ NA, not available.

**Fig 1:**
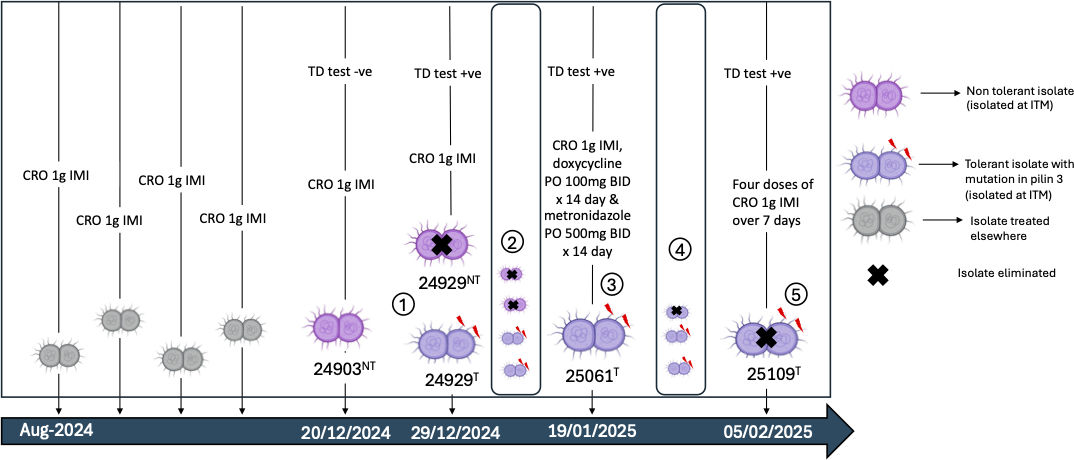
Timeline and treatment history of *N. gonorrhoeae* isolates from August 2024 to February 2025, showing strain emergence, tolerance phenotype, and clearance with prolonged ceftriaxone therapy. (1) Emergence of tolerance during treatment: following ceftriaxone (CRO) treatment, non-tolerant strains (lilac) are killed, while the tolerant strain (purple) survives (TD test + ve). (2) The tolerant phenotype becomes dominant. (3) Phase variation-driven re-growth of a dormant tolerant phenotype: a previously non-dominant tolerant strain grows due to stochastic phase variation events, and this corresponds to the purple phenotype isolate which behaves like a non-tolerant population. (4) Partial clearance under treatment: combined therapy (CRO, doxycycline, and metronidazole) eliminates some isolates, but dormant tolerant populations persist. (5) Final clearance after prolonged ceftriaxone therapy: a 7-day CRO treatment regimen (four doses) results in complete clearance of the tolerant *N. gonorrhoeae* population. Note: NT = non-tolerant; T = olerant; TD = tolerance detection test; IMI = intramuscular injection; BID = twice daily; PO = per os (oral administration).

All isolates were routinely cultured on GC agar supplemented with IsoVitalex enrichment (BD, Franklin Lakes, NJ, USA) and incubated at 36°C in a CO₂ incubator for 18–24 hours. For all assays requiring tolerant subpopulations, fresh colonies were induced from isolate 24929 using the TD test and immediately used. Table 1 summarizes information, including isolation date, ceftriaxone MIC, tolerance phenotype, and available molecular typing data. For further details of the case, please see Kanesaka et al.’s study (9). The timeline of isolate collection, tolerance emergence, and clinical treatment is illustrated in Fig. 1, highlighting the dynamic interplay between treatment pressure and phenotypic switching in *N. gonorrhoeae* during this case.

### Growth curve assay

Bacterial variants (24929-non-tolerant and 24929-tolerant) were suspended in GC broth supplemented with IsoVitaleX at a final concentration of 10³ colony-forming unit (CFU)/mL and incubated at 36°C in a 6% CO₂ atmosphere under static conditions. Colony counts were determined at 0, 2, 4, 6, and 8 hours after incubation by plating 100-fold serial dilutions on GC agar (Becton Dickinson). After incubation at 36°C for 24–48 hours, colonies were counted, and the mean log₁₀ CFU/mL from three replicates was used as the colony count. Results were expressed as the mean ± standard error. For statistical comparisons of doubling times between strains, Student’s *t*-test was applied for each time point. Statistical analyses were conducted using GraphPad Prism version 10 (GraphPad Software, San Diego, CA, USA).

### Quantification of ceftriaxone tolerance using MDK99 assay

The minimum duration for killing 99% of the bacterial population (MDK99) was determined to quantify ceftriaxone tolerance, following a previously described protocol with modifications (14). Three *N. gonorrhoeae* populations were evaluated: (i) tolerant colonies derived from isolate No. 24929, (ii) non-tolerant colonies of isolate No. 24929, and (iii) the initial isolate No. 24903. A bacterial suspension equivalent to a 0.5 McFarland standard (approximately 1 × 10⁸ CFU/mL) was prepared in phosphate-buffered saline (PBS) and serially diluted to achieve a final inoculum of approximately 200 CFU per well. Aliquots were dispensed into 96-well plates (200 µL per well) containing GC broth supplemented in 1% IsoVitalex. The actual inoculum size was confirmed by plating serial dilutions on GC agar and performing colony counts after 24-hour incubation at 36°C in 6% CO₂.

Following ceftriaxone exposure, the bacterial suspension in each well was washed *in situ* three times with sterile PBS (200 µL per wash) to remove residual antibiotic. For each wash, the supernatant was gently aspirated, PBS was added, mixed by gentle pipetting, and the liquid was again removed. After washing, fresh GC broth supplemented with 1% IsoVitaleX (200 µL per well) was added to each well, and the plates were incubated overnight at 36°C in a 6% CO₂ atmosphere before visual inspection for regrowth. This procedure is equivalent in principle to the antibiotic removal step described by Brauner et al. (14) for MDK determination. The MDK99 was defined as the shortest exposure duration after which bacterial regrowth was not observed in any well. Tolerant subpopulations were therefore identified phenotypically, based on the presence of regrowth after antibiotic exposure in the TD test and MDK99 assay, rather than by detection of stable genetic markers. For each condition, three independent experiments were performed. Results were expressed as mean ± standard deviation (SD). For statistical comparisons of MDK99 values between strains, Welch’s *t*-test (unpaired, unequal variance) was applied for each ceftriaxone concentration. Statistical analyses were conducted using GraphPad Prism version 10 (GraphPad Software, San Diego, CA, USA).

### *In vivo* tolerance assay using *G. mellonella*

An *in vivo* tolerance assay was conducted using the *Galleria mellonella* larval infection model to evaluate the survival of ceftriaxone-tolerant *N. gonorrhoeae* strains under antimicrobial pressure (15). Larvae were infected with the strain 24929-tolerant, the strain 24929-non-tolerant, or the initial isolate No. 24903. Bacterial suspensions were adjusted to an 8 McFarland turbidity standard, and 30 µL of the suspension (approximately 2.4 × 10^9^ CFU/mL) was injected into the hemocoel of each larva via the last left proleg using a Hamilton syringe. Ten minutes after infection, ceftriaxone was administered by injecting 10 µL of a 0.016 µg/mL solution into the right proleg. The ceftriaxone concentration (0.016 µg/mL) was selected because it lies within the *in vitro* MIC range of the isolates (0.008–0.016 µg/mL) and produced the strongest tolerance-associated regrowth in the TD test, providing the greatest dynamic range to detect persistence differences between strains. Infected larvae were incubated at 36°C. At 2, 4, 6, and 8 hours post-infection, approximately 50 µL of hemolymph was collected from each larva, serially diluted, and plated on GC selective agar for colony enumeration after incubation at 36°C for 24–48 hours. Recovered colonies were identified as *N. gonorrhoeae* using matrix-assisted laser desorption/ionization time-of-flight mass spectrometry. Each experimental condition was performed in triplicate. Differences in bacterial counts between strains were analyzed at each time point using the Mann-Whitney *U* test. Statistical analyses were conducted using GraphPad Prism version 10 (GraphPad Software, San Diego, CA, USA).

### WGS and bioinformatic analysis

- Illumina sequencing, read processing, and assembly were as follows. The isolates (24929-Non-tolerant, 24929-Tolerant, 25061, and 25109) were outsourced to Eurofins Genomics (Germany) and ZAS (Belgium) for DNA isolation, library preparation, and sequencing. DNA libraries were prepared using the Stranded TruSeq DNA library preparation kit (Illumina Inc., San Diego, CA, USA). Sequencing was performed on a NextSeq 6000 platform using v2 chemistry to generate 2 × 150 bp paired-end reads. Raw Illumina reads were subjected to quality control using FastQC (v0.11.9) (16). Trimming of the raw reads was performed using Trimmomatic (v0.39) with the following parameters: leading and trailing base quality cutoff of 3, sliding window trimming with average quality 15 across four bases, and minimum read length of 30 bases (17). The trimmed raw reads were *de novo* assembled using SPAdes (v3.14.0) with the parameters—trim --depth 150 –careful (18).
- Nanopore sequencing, read processing, and assembly were as follows. Two isolates (24929-non-tolerant and 24929-tolerant isolate) were used. DNA isolation was carried out using MagAttract HMW DNA kit (Qiagen) and sequenced using Oxford Nanopore Technologies (Rapid Barcoding kit SQK-RBK114.24 on R10.4.1 flow cells followed by basecalling and QC via Dorado and NanoPlot). Nanopore sequencing reads for isolate 24929, including both tolerant and non-tolerant subpopulations, were preprocessed using Filtlong (v0.2.1) with Illumina reads provided for guidance. Nanopore reads were filtered by retaining the top 90% of high-quality long reads with a minimum length threshold of 1,000 bp and a target base count of 500 Mbp. Filtered Nanopore reads were assembled using Trycycler (v0.5.5), which integrates multiple long-read assemblers and consensus building steps (19). Initially, reads were subsampled using trycycler with a genome size estimate of 2.3 Mb, and clustering was performed across read subsets. Assembly clusters were generated independently using Flye (20), Raven, and Miniasm/Minipolish (21, 22). Cluster reconciliation involved multiple sequence alignment (trycycler msa) and error correction (trycycler reconcile). Poor-quality or divergent clusters were manually excluded from consensus generation. Final consensus sequences were built using trycycler consensus and concatenated to produce a draft assembly. To further refine the consensus genome, three polishing stages were performed. First, Medaka (1.11.3) was used to polish individual cluster-level assemblies using the Oxford Nanopore model r104_e81_sup_g610 (23). The resulting consensus sequences were then polished with Polypolish (0.6.0), which utilized bwa mem to align paired-end Illumina reads to the assembly. SAM files were filtered using polypolish filter, and polypolish polish was applied to incorporate short-read-based corrections. Finally, the genome was subjected to a third round of polishing using POLCA (MaSuRCA v4.0.8) (24) to correct any residual base-calling errors. The corrected FASTA file was post-processed using seqtk to restore proper contig ordering and formatting.

### Genome annotation, sequence typing, SNP analysis, and comparative genomics

- Genome annotation was as follows. All the draft genomes were annotated using Prokka (v1.14.6) (25). The Nanopore assembly was used as the reference for downstream variant calling and transcriptomic analyses.
- Sequence typing and antimicrobial resistance (AMR) prediction were as follows. All the draft genomes were uploaded to Pathogenwatch (v3.0.2) for *in silico* genotyping and AMR prediction. Sequence types were determined using MLST (PubMLST), NG-STAR, and NG-MAST schemes.
- Comparative genomic analysis was as follows. The tolerant and non-tolerant subpopulations of isolate 24929 were aligned to identify structural differences and single-nucleotide variants associated with ceftriaxone tolerance. Mauve (version 20150226) (26) was employed for multiple genome alignment and to detect any large-scale rearrangements, insertions, deletions, and locally collinear blocks between the tolerant and non-tolerant assemblies. In parallel, for variant analysis, trimmed Illumina reads from isolates 24929-tolerant, 25061, and 25109 were mapped against the baseline Nanopore assembly of isolate 24929-non-tolerant using BWA MEM and single-nucleotide polymorphisms (SNPs) were determined using freebayes implemented in Snippy (v4.6.0) with default parameters (10 × minimum read coverage and 90% read concordance at the variant locus).

### RNA-seq and bioinformatic analysis

RNA-seq was carried out as previously described in Manoharan-Basil et al. (7). Briefly, two *N. gonorrhoeae* isolates—the ceftriaxone-tolerant isolate 24929 and the isogenic non-tolerant isolate—were recovered after 24 hours of growth on GC-chocolate agar (Becton Dickinson) and stored in RNAlater (Invitrogen) at −80°C, prior to processing. As only one biological sample per condition was analyzed, biological replication was not available, and the resulting transcriptomic differences were interpreted as candidate differentially expressed genes (DEGs). The isolates were outsourced to Eurofins Genomics (Germany) where total RNA was isolated followed by ribodepletion to remove rRNA. The Stranded TruSeq RNA Library Preparation Kit was used for cDNA library preparation, and the library was then sequenced on a NextSeq6000, v2, 1 × 150 bp platform (Illumina Inc). The raw reads were analyzed using CLC Genomics Workbench v22 (Clcbio, Denmark), according to Liu and Di (27). Reads were quality-checked and mapped to the respective draft genomes using CLC Genomics Workbench v20 (Qiagen). Transcript abundance was estimated as transcripts per million (TPM) and raw read counts per gene. The resulting expression matrices were exported from CLC and analyzed in RStudio (2025.05.1) using a customized pipeline.

Differential expression analysis was conducted using two complementary approaches. First, TPM values were used to calculate log₂ fold changes (FCs) between the tolerant and non-tolerant isolates, with DEGs defined as those with log₂ FC ≥ 1 and TPM > 5 in at least one sample. Second, raw gene-level counts were analyzed using edgeR (Version 4.6.2). Because only one sample per condition was available, a fixed-dispersion sensitivity analysis was conducted using a range of biological dispersion values (0.01, 0.05, 0.1, 0.2, and 0.4). DEGs were retained if they exhibited a false discovery rate (FDR) < 0.05 at each dispersion threshold. Final candidate DEGs were defined as those meeting both statistical significance (FDR < 0.05 at dispersion = 0.1) and FC criteria. Only genes identified as DEGs by both methods were included in the final DEG set used for interpretation and downstream visualization. Data visualization, including heatmaps of selected DEGs, was performed using the ggplot2 and heatmap packages in R. To further explore the functional relationships among DEGs, protein-protein interaction network analysis was performed using the STRING database (28).

## RESULTS

### Growth curves of tolerant and non-tolerant strains

**Fig 2:**
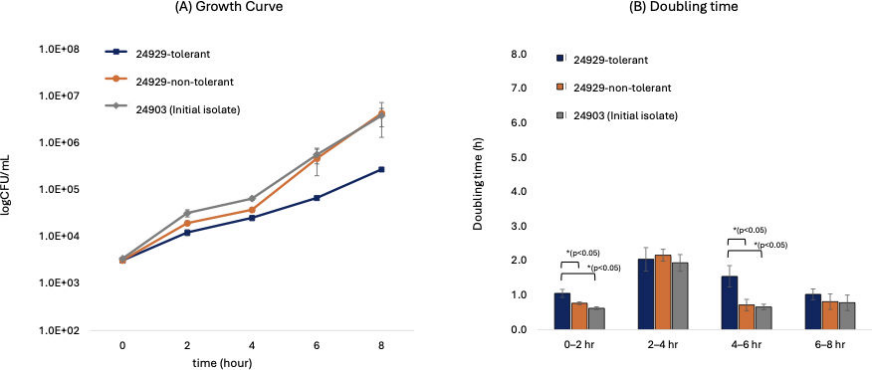
Growth curves and doubling time analysis of *N. gonorrhoeae* strains. (**A**) Growth curves of 24929-tolerant and 24929-non-tolerant and 24903 (initial isolate) strains in GC broth. Bacterial growth was monitored by measuring CFU at indicated time points, and values are shown as log₁₀ CFU/mL. Data represent mean ± SD of three independent experiments.(**B**) Doubling times of each strain were calculated for four different intervals based on the growth curve data shown in panel **A**. Error bars indicate SD from three biological replicates. *, *P* < 0.05; **, *P* < 0.01; ***, *P* < 0.001.

All strains entered logarithmic growth. Differences in bacterial counts were observed from 2 hours, with the strain 24929-tolerant showing lower bacterial counts compared to the non-tolerant strains. The difference became more pronounced at 6 and 8 hours, where strain 24929-tolerant showed approximately one log₁₀ CFU/mL lower counts than the non-tolerant isogenic variant and the initial isolate No. 24903 (Fig. 2).

### Quantification of ceftriaxone tolerance using MDK99

**Fig 3:**
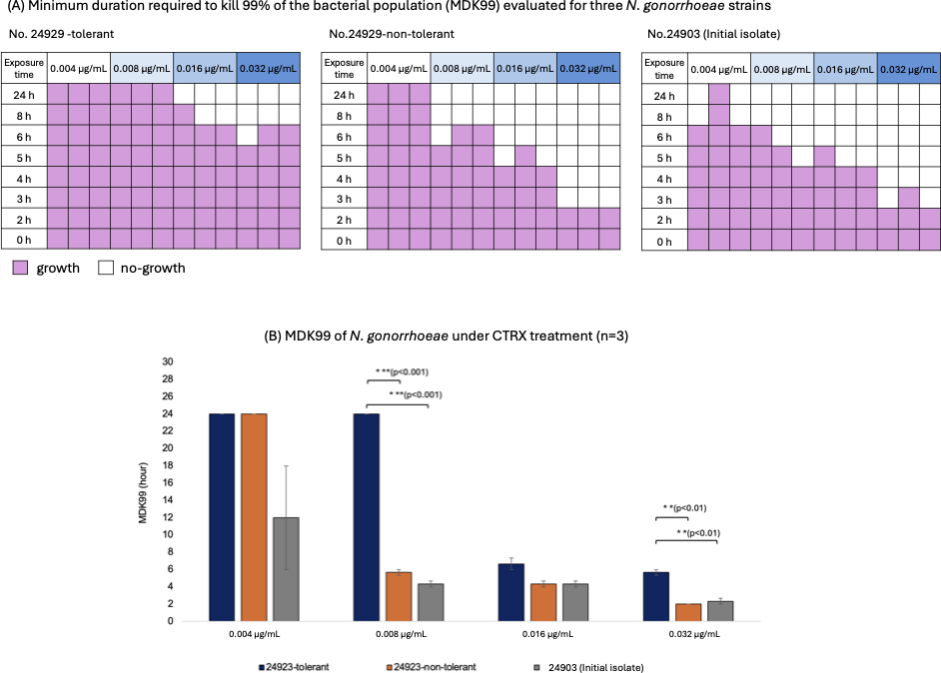
MDK99 of *N. gonorrhoeae* under ceftriaxone treatment. (**A**) Minimum duration required to kill 99% of the bacterial population (MDK99) evaluated for three *N. gonorrhoeae* strains (24929-tolerant, 24929-non-tolerant, and 24903 initial isolate) exposed to ceftriaxone at concentrations ranging from 0.004 to 0.032 µg/mL. Pink squares indicate conditions where regrowth was observed; white squares indicate no detectable growth. (**B**) Mean MDK99 values ± SD from three independent experiments, shown as bar graphs. *, *P* < 0.05; **, *P* < 0.01; ***, *P* < 0.001.

The MDK99 values were determined for all strains across a range of ceftriaxone concentrations (Fig. 3A and B). The 24929-tolerant strain exhibited persistent survival at lower drug concentrations with MDK99 values of 24 hours at both 0.004 and 0.008 µg/mL. At higher concentrations, 0.016 and 0.032 µg/mL, the MDK99 values decreased to 7 and 6 hours, respectively. In contrast, the isogenic 24929-non-tolerant variant showed faster killing kinetics with MDK99 values of 24 hours at 0.004 µg/mL, 6 hours at 0.008 µg/mL, 4 hours at 0.016 µg/mL, and 2 hours at 0.032 µg/mL. The initial isolate (No. 24903), which did not exhibit ceftriaxone tolerance, displayed MDK99 values of 12 hours at 0.004 µg/mL, 4 hours at 0.008 µg/mL and 0.016 µg/mL, and 2 hours at 0.032 µg/mL.

Statistically significant differences in MDK99 were observed between the 24929-tolerant strain and the 24929-non-tolerant variant at 0.008 µg/mL (*P* = 0.0003) and 0.032 µg/mL (*P* = 0.0082). Similarly, significant differences were found between the 24929-tolerant strain and the initial isolate No. 24903 at 0.008 µg/mL (*P* = 0.0003) and 0.032 µg/mL (*P* = 0.0021).

### Persistence of tolerant strain in the *G. mellonella* infection model under ceftriaxone treatment

**Fig 4:**
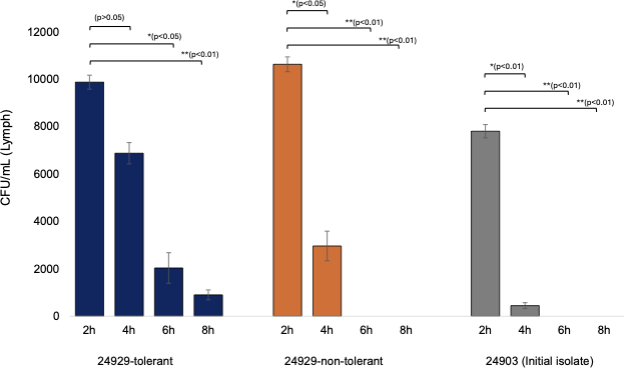
Survival of *N. gonorrhoeae* strains in larvae following ceftriaxone (CTRX) treatment (0.016 µg/mL). *G. mellonella* larvae were infected with *N. gonorrhoeae* strains 24929-tolerant and 24929-non-tolerant and 24903 (initial isolate), followed by injection of ceftriaxone (0.016 µg/mL) 10 minutes post-infection. Bacterial load in the hemolymph was quantified at 2, 4, 6, and 8 hours post-treatment by plating on GC selective agar. Each bar represents mean CFU/mL from individual larvae (*n* = 1–3) at each time point. Error bars indicate SD. *, *P* < 0.05; **, *P* < 0.01; ***, *P* < 0.001.

After ceftriaxone treatment, both the non-tolerant strain No. 24929 and the initial isolate No. 24903 showed bacterial counts below the detection limit at 6 and 8 hours post-infection. In contrast, the 24929-tolerant strain remained detectable at both time points, with average counts of approximately 2.0 × 10^3^ CFU/mL at 6 hours and approximately 9.0 × 10^2^ CFU/mL at 8 hours. Figure 4 shows within-strain changes in bacterial counts over time, following ceftriaxone exposure in the *G. mellonella* model. Significant reductions in CFU were observed between sequential time points within each strain, confirming that ceftriaxone progressively reduced bacterial burden. The tolerant isolate (24929) exhibited a slower decline than the non-tolerant and initial isolates, consistent with its prolonged survival phenotype.

### Whole-genome assembly and comparative genomics

Hybrid assembly of isolate 24929 (non-tolerant and tolerant) using long- and short-read sequencing produced a high-quality genome with minimal fragmentation. The final assembly of the 24929-non-tolerant and 24929-tolerant isolates consisted of a single circular chromosome of approximately 2,227,584 and 2,227,201 bp, respectively. Comparative alignment using Mauve revealed high structural similarity between the 24929-tolerant and 24929-non-tolerant genomes. No large-scale chromosomal rearrangements were detected. However, localized deletions were observed in a region encoding fimbrial proteins (*pilE_3*).

### Sequence typing, AMR, and SNP profiling

Whole-genome sequencing (WGS) and sequence-based typing were conducted for isolates that were TD test positive, as these were the focus of the tolerance-related genomic analysis. Accordingly, the initial isolate 24903 that was TD-negative was not included in the WGS data set (Table 1) and all other isolates (24929, 25061, and 25109), including the tolerant and non-tolerant subpopulations of isolate 24929, belonged to the same sequence type (ST7822) according to the PubMLST schema (Table 1). NG-STAR typing assigned all four of these genomes to ST5423, with identical profiles across *penA* (5.002), *mtrR* (597), *porB* (14), *ponA* (1), *gyrA* (7), *parC* (3), and 23S rRNA (100). NG-MAST analysis showed no discriminatory power, as *por* alleles were non-typeable (“new”) and *tbpB* alleles were all 33.

PubMLST/PathogenWatch predicted AMR profiles were consistent across all isolates, with resistance noted for azithromycin, ciprofloxacin, and sulfonamides, and intermediate susceptibility predicted for penicillin and tetracycline. No ceftriaxone or cefixime resistance determinants were detected, aligning with phenotypic MIC data (Table 1). Resistance-associated mutations included canonical substitutions in the fluoroquinolone resistance determinants GyrA (S91F and D95A) and ParC (S87R), as well as FolP (R228S), which is associated with sulfonamide resistance. Additionally, a non-mosaic *penA* allele classified as type V, with additional mutations—Ala517Gly and Gly543Ser, alongside ponA Leu421Pro, a substitution linked to decreased susceptibility to β-lactams (Table S1). RpsJ Val57Met substitution, associated with reduced susceptibility to tetracycline, was detected. The presence of the *mtrD* mosaic allele and *mtrR* promoter mosaic variant, both of which are linked to enhanced efflux-mediated resistance to macrolides, was observed. Importantly, these mutations were identical between the tolerant and non-tolerant isolates.

SNP analysis across the three isolates revealed distinct yet overlapping mutational profiles, primarily affecting fimbrial and functional genes (Table S1). In the ceftriaxone-tolerant subpopulation of isolate 24929, six non-synonymous mutations were identified exclusively within the *pilE_3* gene, which encodes a type IV fimbrial protein (Table S1). These included amino acid substitutions such as Thr58Lys, Asn60Lys, Asn66Lys, and Lys72Arg, as well as indels 185_188delGCAAinsACGG and 205_209delAAAGAinsCAAG resulting in substitutions at positions 62 (GlyAsn62AspGly) and 69 (LysAsp69GlnGly), respectively.

Isolate 25061 exhibited an identical *pilE_3* mutation profile to 24929-tolerant, indicating close genetic relatedness. In contrast, isolate 25109 demonstrated a more diverse SNP profile. In addition to the same *pilE_3* mutations observed in 24929-tolerant and 25061, this isolate harbored three additional variants. A disruptive in-frame deletion (354_365delTACGCTGGAAGC) in a hypothetical protein was identified. A missense mutation (p.Pro456Ser) in *parE*, encoding DNA topoisomerase IV subunit B, was present and the isolate had a CIP MIC of >32 µg/mL (Table 1). Furthermore, a Phe49Ser substitution was detected in *tbpB*, a gene involved in transferrin-mediated iron uptake and host colonization. Finally, a distinct SNP in *pilE_6* (Thr52Gln) was also observed, indicating further structural modification of fimbrial components.

### Transcriptomic changes associated with tolerance

Transcriptomic profiling of the 24929-tolerant isolate compared to its non-tolerant isogenic variant revealed a distinct gene expression signature associated with the tolerance phenotype. Several pilin-associated genes, including *pilE_1*, *pilE_3*, *pilE1_2*, and *pilE_5*, were consistently upregulated in the tolerant isolate (Table S2).

In contrast, genes involved in ribosome function and metal-responsive regulation were downregulated in the tolerant isolate. Specifically, *rpmE2* and *ykgO* (*rmpJ*), which encode zinc-independent paralogs of ribosomal proteins L31 and L36, respectively, showed decreased expression.

**Fig 5:**
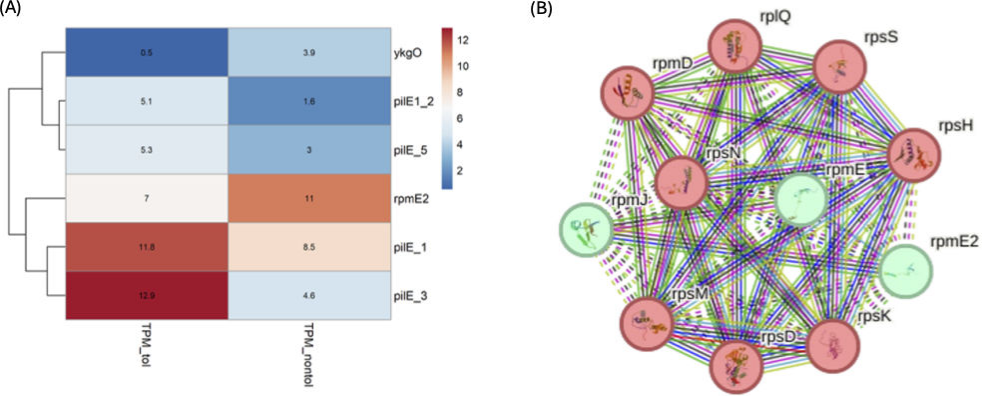
(**A**) Heatmap of DEGs between 24929-tolerant vs 24929-non-tolerant *N. gonorrhoeae*. Upregulated pilin genes (pilE_1, pilE_3, pilE1_2, and pilE_5) and downregulated ribosomal paralogs (rpmE2 and ykgO) are shown. Color intensity corresponds to expression levels, with red indicating higher expression and blue indicating lower expression (in FC in expression). (**B**) STRING network of rpmE2 and ykgO (rpmJ) in *N. gonorrhoeae*. Protein-protein interaction network generated using STRING showing interactions. Downregulated zinc-independent ribosomal paralogs (rpmE2 and rpmJ) are highlighted in green, clustering with core ribosomal components (rps and rpm) shown in pink. The network illustrates functional connectivity. Edge colors indicate known (green), predicted (blue), and co-expression (purple) relationships.

STRING analysis revealed that the downregulated genes such as *rpmE2* and *ykgO* clustered within the translation machinery module (Fig. 5A and B).

### Summary of findings and conceptual model

Taken together, these results suggest that ceftriaxone tolerance in *N. gonorrhoeae* is associated with multiple phenotypic changes, including delayed killing kinetics (MDK99), enhanced persistence *in vivo*, pilin gene upregulation, ribosomal gene downregulation, and specific *pilE_3* mutations. While delayed killing and *in vivo* persistence represent phenotypic manifestations of tolerance, the accompanying molecular signatures likely reflect adaptive responses that support this phenotype. To visually integrate these findings, we developed a conceptual model summarizing the proposed mechanisms contributing to ceftriaxone tolerance (Fig. S1).

## DISCUSSION

In this study, we provided the first characterization of a ceftriaxone-tolerant clinical isolate of *N. gonorrhoeae*, using integrated genomic and transcriptomic profiling by direct comparison of tolerant with non-tolerant isogenic strains derived from the clinical isolate. Despite identical ceftriaxone MICs (0.008 µg/mL), the tolerant and non-tolerant variants of isolate 24929 exhibited distinct molecular features. The ceftriaxone-tolerant strain (No. 24929) exhibited distinct phenotypic behaviors, including altered growth kinetics, prolonged survival under antibiotic exposure (MDK99), and enhanced persistence in the *G. mellonella in vivo* infection model compared to non-tolerant strains. Collectively, our findings suggest that tolerance may contribute to recurrent gonococcal infections and treatment failures. The fact that the sequence types from his fifth and sixth episodes of gonorrhea were different reiterates the importance of reinfection as a common cause of apparent treatment failure.

Although the mechanisms of AMR in *N. gonorrhoeae* have been extensively studied, the prevalence, mechanisms, and implications of bacterial tolerance have remained largely unexplored (3, 4, 7). Our results suggest that, even if isolates are classified as susceptible based on MIC values, the presence of tolerance, which is a transient and non-heritable phenotype, allows bacterial populations to survive prolonged antibiotic exposure. In particular, the MDK99 experiments revealed that the tolerant isolate remained viable for at least 24 hours under exposure to ceftriaxone at low concentrations (0.004 and 0.008 µg/mL), highlighting the possibility that tolerance might require prolonged antimicrobial therapy. Importantly, tolerance is not detected by standard AST, thereby representing a diagnostic blind spot that may contribute to treatment failures. Specific methodologies, such as the TD test, may be useful to identify these phenotypes (3, 4).

Unsurprisingly, the tolerant strain demonstrated a fitness cost in terms of reduced growth rate compared to non-tolerant counterparts, particularly evident in its longer doubling time during the initial 0 to 2 hours of culture. While the precise cause of this initial delay remains unclear, similar growth lags have been reported in other bacterial species and are sometimes associated with shifts in metabolic activity or transient activation of stress-related pathways (29, 30). For example, toxin-antitoxin systems or the stringent response have been shown to modulate growth dynamics in response to stress in *E. coli* and *S. aureus* (29, 31). It is therefore plausible, although unconfirmed in our model, that such mechanisms may contribute to the early-phase growth delay observed in the ceftriaxone-tolerant *N. gonorrhoeae* strain. Further investigation is needed to clarify whether tolerance in this context involves similar physiological trade-offs. However, the significant advantage of prolonged survival under antibiotic exposure observed *in vitro* and in the *G. mellonella* model suggests that any fitness disadvantages in untreated conditions are outweighed by the selective advantage under antibiotic treatment scenarios. This phenomenon may facilitate the persistence and eventual emergence of resistant strains, especially during suboptimal antibiotic treatments or in cases of inadequate antibiotic penetration into infection sites. Similar observations have been reported in other pathogens such as *S. aureus* and *E. coli*, where tolerance provides a reservoir from which resistance can subsequently emerge under antibiotic selection pressure (3–5).

*In vivo* assessments using the *G. mellonella* model further underscored the clinical relevance of tolerance. The tolerant strain exhibited significantly enhanced persistence compared to non-tolerant strains after ceftriaxone treatment. These results suggest that tolerance could explain some of the rare reports of recurrent or persistent infection observed clinically despite appropriate antibiotic therapy. Additionally, these tolerant isolates may be transmitted to sexual partners. These findings highlight the necessity to include tolerance as a possible explanation for treatment failure in gonococcal infections (2, 3, 32). Although the *G. mellonella* model does not reproduce the human urogenital environment, it provides a practical proof of principle platform for comparing persistence associated with tolerance across isolates.

WGS revealed high structural similarity between the 24929-tolerant and 24929-non-tolerant genomes, with no evidence of large-scale chromosomal rearrangements. However, localized regions of low homology were identified, mainly within the fimbrial gene *pilE_3*, consistent with a model in which *pilE_3* variation may contribute to phenotypic divergence. If these pilin variations do not stem from phase variations, then the consistent presence of *pilE_3* mutations across all tolerant-associated isolates, including isolate 25061, may mean that structural variation in pili may play a role in mediating tolerance. Although MDK99 quantification was not performed for 25061, the presence of identical *pilE_3* mutations and a positive TD test suggests that it may share similar tolerance-associated survival under antimicrobial exposure. To confirm whether *pilE_3* variation and pilin gene upregulation directly drive the tolerant phenotype, future work using reverse genetics (knock-out and complementation approaches) will be required.

Type IV pili are outer membrane structures that are crucial for mediating initial cellular adherence, natural transformation competence, twitching motility, and immune evasion through antigenic variation and phase variation (33–37). Mutations in *pilE_3*, including multiple non-synonymous substitutions and indels, are predicted to alter protein structure and surface presentation. Such changes may modulate interactions with host tissues, impact biofilm formation, and reduce antibiotic permeability, collectively enhancing bacterial persistence without elevating MICs. These findings are further supported by transcriptomic data, which revealed upregulation of multiple pilin-associated genes (*pilE_1*, *pilE_3*, *pilE1_2*, and *pilE_5*) in the tolerant subpopulation. This is consistent with enhanced pilus biogenesis and supports a model of surface remodeling as a contributor to tolerance.

In contrast to the upregulation of pilus-related genes, genes involved in ribosome biogenesis and metal-responsive regulation were downregulated in the tolerant isolate. Notably, *rpmE2* and *ykgO*, which are paralogs of zinc-independent ribosomal proteins L31 and L36, respectively, were repressed. Unlike L31 and L36, these paralogs do not contain a Zn-binding motif. In *E. coli* L31 and its paralog YkgM are encoded by *rpmE* and *ykgM*, respectively, while L36 and its paralog YkgO are encoded by *rpmJ* and *ykgO*, respectively (38). Their downregulation may indicate a shift toward ribosome hibernation or translational suppression, which are commonly associated with bacterial persistence. Studies in *E. coli*, *Pseudomonas aeruginosa*, and *M. tuberculosis* have shown that disruption of zinc homeostasis and ribosomal switching contribute to antimicrobial tolerance and virulence (39–42). STRING analysis showed that the downregulated ribosomal paralogs clustered within the translation machinery module, indicating coordinated suppression of protein synthesis.

These findings align with our earlier study, in which ceftriaxone tolerance was experimentally induced in *N. gonorrhoeae* through prolonged exposure to subinhibitory concentrations of the drug. Transcriptomic analysis revealed no DEGs at the first time point (day 1), but after 3 days of intermittent ceftriaxone exposure, 13 genes were significantly downregulated, including tRNA-Ser (C7S06_RS03100), tRNA-Leu (C7S06_RS04945), and ribosomal RNA genes (16S and 23S rRNA). By day 7, 51 genes were differentially expressed, predominantly downregulated, including secB (encoding the protein-export chaperone SecB), additional tRNAs, and rRNA operon components. Collectively, both studies support a model in which tolerance is mediated through ribosome remodeling and translational shutdown, possibly as a survival strategy under sustained antimicrobial pressure. The overlap in regulatory response, despite differing experimental contexts, reinforces the biological plausibility of this tolerance mechanism and highlights the relevance of ribosomal regulation as a consistent signature of ceftriaxone tolerance in *N. gonorrhoeae*.

There was little difference in resistance-associated mutations between isolates. All the isolates had canonical mutations in *gyrA*, *parC*, *folP*, and *penA*, as well as mosaic *mtr* alleles indicative of multidrug resistance. Isolate 25061 did, however, acquire an additional mutation Pro456Ser in *parC*, that is outside the quinolone resistance determining region, which was however associated with a markedly elevated ciprofloxacin MIC of 32 µg/mL, suggesting potential additive effects of non-canonical mutations on fluoroquinolone susceptibility. This isolate also had a substitution in TbpB (Phe49Ser). Given that all isolates carried *mtrD* and *mtrR* promoter mosaics as well as *penA* substitutions (A517G/G543S), tolerance may have arisen on a pre-resistant genetic background with reduced intracellular antibiotic exposure. Whether similar MDK99 prolongation would occur in *mtr*-wild-type strains remains to be determined.

The presence of tolerance might facilitate ongoing transmission within populations, exacerbating the public health burden of gonorrhea and possibly accelerating the emergence of resistant strains through prolonged bacterial survival during antibiotic exposure. We hypothesize that tolerance may play an underestimated role in shaping antibiotic resistance epidemiology, potentially contributing to the emergence of multidrug-resistant gonococcal strains through prolonged sublethal antibiotic exposure in clinical and community settings. This extended bacterial survival time might allow for genetic diversification, increasing the likelihood of acquiring resistance mutations. Future studies should investigate the genetic stability and mutation rates of tolerant populations under antibiotic exposure to further substantiate this hypothesis.

Furthermore, the precise mechanisms underlying the enhanced *in vivo* survival of tolerant strains remain speculative. One plausible explanation is the induction of stress response pathways, such as toxin-antitoxin systems, dormancy induction, or alterations in metabolic activity, allowing bacteria to persist under antibiotic stress conditions (29). Additionally, structural modifications such as cell wall thickening or biofilm formation may contribute to tolerance by physically reducing antibiotic penetration or efficacy (3). These possibilities warrant further exploration using advanced transcriptomic, proteomic, and microscopic analyses to clarify tolerance mechanisms at a molecular and cellular level.

A promising future perspective involves evaluating whether zinc supplementation can reverse ceftriaxone tolerance. In *Klebsiella pneumoniae*, zinc supplementation reversed tigecycline resistance by interfering with ribosomal remodeling and membrane permeability mechanisms (43). This study showed that increasing intracellular zinc levels using zinc ionophores such as PBT2 can suppress superoxide dismutase activity, elevate reactive oxygen species, and inhibit cell wall synthesis, ultimately interfering with efflux pump function and enhancing susceptibility to antibiotics (43). Testing whether zinc co-treatment accelerates ceftriaxone-mediated killing in a *G. mellonella* infection model could offer a new strategy to counteract tolerance and improve treatment outcomes.

This work is based on four isolates recovered from a single patient, a transgender man receiving gender-affirming testosterone therapy. Testosterone can modify vaginal physiology and the microbiome, which may in turn influence gonococcal gene expression and antibiotic penetration; our results should therefore be interpreted with this biological context in mind. In addition, RNA-seq in this study used one biological sample per condition, limiting statistical replication (see Materials and Methods and Table S2). Consequently, the generalizability of *pilE_3* variation and ribosome-related transcriptional changes as markers of ceftriaxone tolerance remains uncertain. Larger, anatomically diverse cohorts, including individuals not receiving hormone therapy, are needed, to test whether these associations hold broadly.

In conclusion, our study highlights ceftriaxone tolerance in *N. gonorrhoeae* as a clinically relevant phenomenon that contributes to bacterial persistence and may underlie unexplained treatment failures. The current reliance on MIC-based definitions of susceptibility overlooks this tolerance phenotype. Our findings, therefore, suggest the need to consider assessing for gonococcal tolerance in episodes of treatment failure. Implementation of the TD test alongside routine susceptibility testing may help identify tolerance as an early warning sign of treatment failure and resistance emergence. These steps may assist in addressing the global threat posed by drug-resistant gonorrhea.

## Data Availability

The raw reads are available under NCBI BioProject PRJNA1287587.

## SUPPLEMENTAL MATERIAL

Figure S1 msystems.01298-25-s0001.pdf Transcriptomic and phenotypic remodeling associated with ceftriaxone tolerance in *N. gonorrhoeae.*

Table S1 msystems.01298-25-s0002.docx Identified genomic variants in *N. gonorrhoeae isolates.*

Table S2 msystems.01298-25-s0003.docx Candidate DEGs identified in ceftriaxone-tolerant vs. non-tolerant *N. gonorrhoeae.*

ASM does not own the copyrights to Supplemental Material that may be linked to, or accessed through, an article. The authors have granted ASM a non-exclusive, world-wide license to publish the Supplemental Material files. Please contact the corresponding author directly for reuse.
