# Supplementary material for: Characterizing ceftriaxone tolerance in Neisseria gonorrhoeae across in vitro and in vivo models
Source: mSystems. 2026 Jan 8;11(2):e01298-25. doi: 10.1128/msystems.01298-25 (PMC12911389; doi:10.1128/msystems.01298-25)
Supplement: Figure S1 — Transcriptomic and phenotypic remodeling associated with ceftriaxone tolerance in N. gonorrhoeae. [file msystems.01298-25-s0001.pdf]

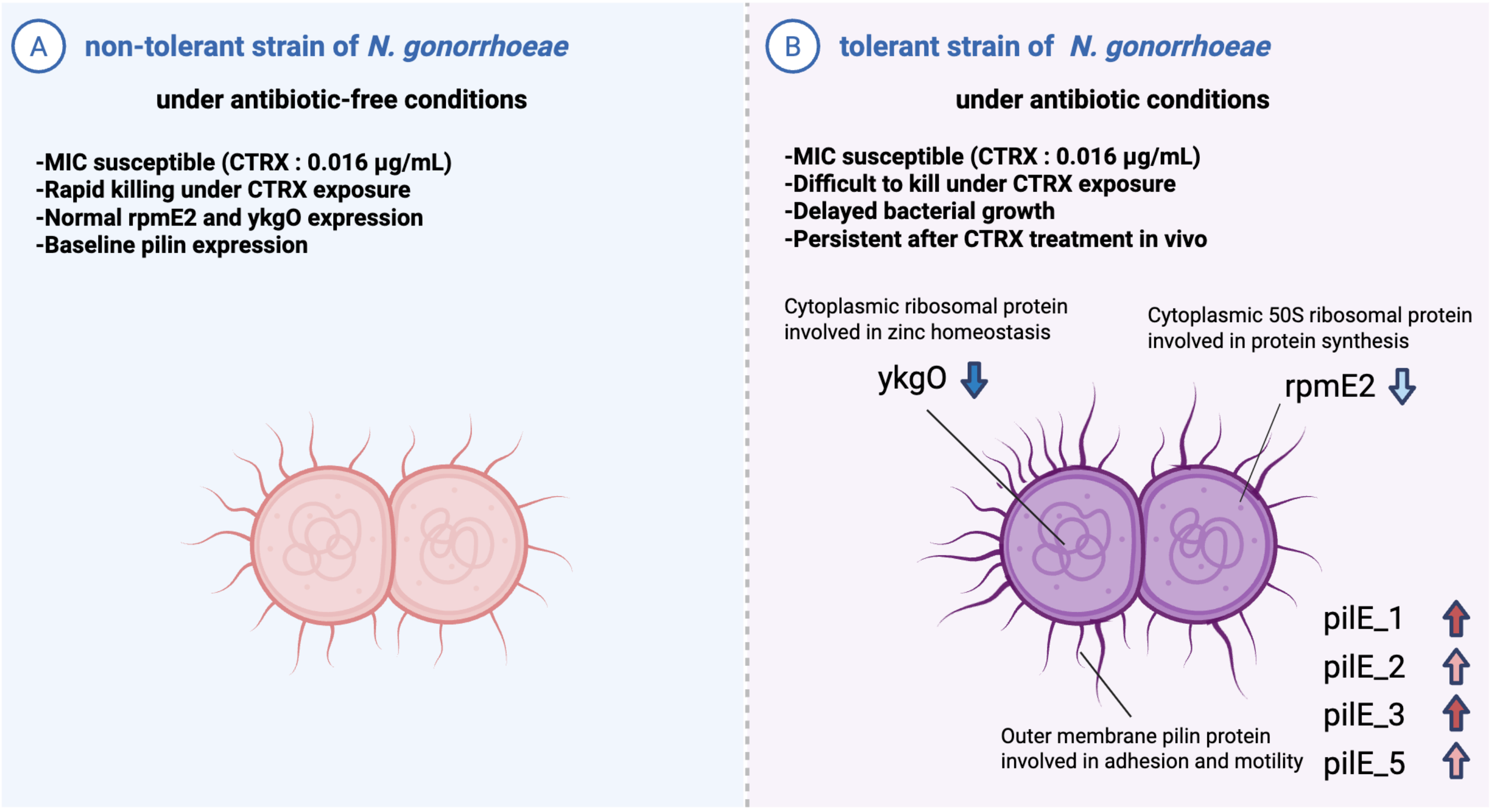

Figure S1. Transcriptomic and phenotypic remodeling associated with ceftriaxone tolerance in *Neisseria gonorrhoeae*.
