# Supplementary material for: Characterizing ceftriaxone tolerance in Neisseria gonorrhoeae across in vitro and in vivo models
Source: mSystems. 2026 Jan 8;11(2):e01298-25. doi: 10.1128/msystems.01298-25 (PMC12911389; doi:10.1128/msystems.01298-25)
Supplement: Table S1 — Identified genomic variants in N. gonorrhoeae isolates. [file msystems.01298-25-s0002.docx]

| Table S1. Identified genomic variants in *N. gonorrhoeae* isolates with gene locus protein annotation and SNP details | | | | |
| --- | --- | --- | --- | --- |
| **Isolate id** | **Locus tag** | **Gene** | **Product** | **SNP** |
| 24929_Tolerant | POMBOJNF_01848 | *pilE_3* | Fimbrial protein | missense_variant c.173C>A p.Thr58Lys |
|  |  |  |  | missense_variant c.180T>A p.Asn60Lys |
|  |  |  |  | missense_variant c.185_188delGCAAinsACGG p.GlyAsn62AspGly |
|  |  |  |  | missense_variant c.198T>A p.Asn66Lys |
|  |  |  |  | missense_variant c.205_209delAAAGAinsCAAGG p.LysAsp69GlnGly |
|  |  |  |  | missense_variant c.215A>G p.Lys72Arg |
| 25061 | POMBOJNF_01848 | *pilE_3* | Fimbrial protein | missense_variant c.173C>A p.Thr58Lys |
|  |  |  |  | missense_variant c.180T>A p.Asn60Lys |
|  |  |  |  | missense_variant c.185_188delGCAAinsACGG p.GlyAsn62AspGly |
|  |  |  |  | missense_variant c.198T>A p.Asn66Lys |
|  |  |  |  | missense_variant c.205_209delAAAGAinsCAAGG p.LysAsp69GlnGly |
|  |  |  |  | missense_variant c.215A>G p.Lys72Arg |
| 25109 | POMBOJNF_00464 |  | hypothetical protein | disruptive_inframe_deletion c.354_365delTACGCTGGAAGC p.Thr119_Ala122del |
|  | POMBOJNF_01327 | *parE* | DNA topoisomerase 4 subunit B | missense_variant c.1366C>T p.Pro456Ser |
|  | POMBOJNF_01502 | *tbpB* | Transferrin-binding protein 2 | missense_variant c.146T>C p.Phe49Ser |
|  | POMBOJNF_01848 | *pilE_3* | Fimbrial protein | missense_variant c.173C>A p.Thr58Lys |
|  |  |  |  | missense_variant c.180T>A p.Asn60Lys |
|  |  |  |  | missense_variant c.185_188delGCAAinsACGG p.GlyAsn62AspGly |
|  |  |  |  | missense_variant c.198T>A p.Asn66Lys |
|  |  |  |  | missense_variant c.205_209delAAAGAinsCAAGG p.LysAsp69GlnGly |
|  |  |  |  | missense_variant c.215A>G p.Lys72Arg |
|  | POMBOJNF_01871 | *pilE_6* | Fimbrial protein | missense_variant c.154_155delACinsCA p.Thr52Gln |
