# Supplementary material for: Characterizing ceftriaxone tolerance in Neisseria gonorrhoeae across in vitro and in vivo models
Source: mSystems. 2026 Jan 8;11(2):e01298-25. doi: 10.1128/msystems.01298-25 (PMC12911389; doi:10.1128/msystems.01298-25)
Supplement: Table S2 — Candidate DEGs identified in ceftriaxone-tolerant vs. non-tolerant N. gonorrhoeae. [file msystems.01298-25-s0003.docx]

| Name | Chromosome_tol | Region_tol | Expression value_tol | TPM_tol | RPKM_tol | Gene length_tol | Unique gene reads_tol | Total gene reads_tol | Chromosome_nontol | Region_nontol | Expression value_nontol | TPM_nontol | RPKM_nontol | Gene length_nontol | Unique gene reads_nontol | Total gene reads_nontol | log2FC | abs_log2FC | logFC | logCPM | PValue | FDR | Dispersion | direction |
| --- | --- | --- | --- | --- | --- | --- | --- | --- | --- | --- | --- | --- | --- | --- | --- | --- | --- | --- | --- | --- | --- | --- | --- | --- |
| *pilE_3* | FGHMOFLP_1 | 1787929..1788291 | 7598.758971 | 7598.758971 | 4899.73032 | 363 | 103352 | 103390 | POMBOJNF_1 | 1786681..1787115 | 23.8999563 | 23.8999563 | 14.9668863 | 435 | 74 | 141 | 8.25366664 | 8.25366664 | 7.89257732 | 10.9280711 | 4.04E-19 | 4.60E-16 | 0.1 | Up in Tolerant |
| *rpmE2* | FGHMOFLP_1 | complement(701381..701656) | 123.3423876 | 123.3423876 | 79.5319919 | 276 | 1276 | 1276 | POMBOJNF_1 | complement(701488..701763) | 2060.00784 | 2060.00784 | 1290.04014 | 276 | 7711 | 7711 | -4.0509599 | 4.0509599 | -4.2200726 | 8.87489912 | 1.48E-08 | 8.44E-06 | 0.1 | Up in Non-Tolerant |
| *pilE1_2* | FGHMOFLP_1 | 1786577..1786951 | 32.15717773 | 32.15717773 | 20.7351621 | 375 | 452 | 452 | POMBOJNF_1 | 1788314..1788625 | 2.12693965 | 2.12693965 | 1.3319549 | 312 | 7 | 9 | 3.40649793 | 3.40649793 | 4.01609298 | 3.27322447 | 1.16E-06 | 4.41E-04 | 0.1 | Up in Tolerant |
| *ykgO* | FGHMOFLP_1 | complement(701256..701381) | 0.423477372 | 0.423477372 | 0.27306103 | 126 | 2 | 2 | POMBOJNF_1 | complement(701363..701488) | 14.0445539 | 14.0445539 | 8.79513074 | 126 | 24 | 24 | -3.4017499 | 3.40174986 | -5.0834877 | 0.19501475 | 3.54E-06 | 0.00100647 | 0.1 | Up in Non-Tolerant |
| *pilE_1* | FGHMOFLP_1 | 1783740..1784183 | 3588.876304 | 3588.876304 | 2314.13131 | 444 | 59697 | 59727 | POMBOJNF_1 | 1783844..1784287 | 356.214487 | 356.214487 | 223.072446 | 444 | 2145 | 2145 | 3.32907164 | 3.32907164 | 3.17431216 | 10.2821331 | 7.75E-06 | 0.00176298 | 0.1 | Up in Tolerant |
| *pilE_5* | FGHMOFLP_1 | 1810102..1810518 | 39.60275079 | 39.60275079 | 25.5361171 | 417 | 613 | 619 | POMBOJNF_1 | 1809665..1809961 | 6.95134485 | 6.95134485 | 4.35314552 | 297 | 28 | 28 | 2.35230668 | 2.35230668 | 2.83874536 | 3.79203055 | 1.22E-04 | 0.02319919 | 0.1 | Up in Tolerant |

Table S2 Candidate DEGs identified in ceftriaxone-tolerant vs. non-tolerant *N. gonorrhoeae*
